# Voices From the Frontlines in Longterm Care During COVID-19: Narratives of Direct Care Workers

**DOI:** 10.1093/geroni/igaa057.3515

**Published:** 2020-12-16

**Authors:** Christin Wolf, Andrea Freidus, Dena Shenk

**Affiliations:** University of North Carolina at Charlotte, Charlotte, North Carolina, United States

## Abstract

Our study draws from the narratives of 30 staff caring for residents in congregate care communities in central North Carolina from June-September 2020. It is part of phase 2 of an on-going 3-phase rapid qualitative appraisal of workers providing longterm care to older adults with the purpose of disseminating findings to key stakeholders to inform policy, programming, and funding decisions. The 3-phase project involves semi-structured interviews with 60+ participants that were videorecorded using a web-based platform. We report on the emotional and visceral experiences of these direct care workers providing care during the pandemic. We organize the data into four affect categories: fear/anxiety, sadness/grief, anger/frustration, and trauma/stress. The 30 participants include nurses, activities staff, med techs, CNAs, housekeepers, dining staff, chaplains and administrators at nursing homes, assisted living communities, memory care units and continuing care retirement communities. We amplify the voices of these formal caregivers in order to demonstrate how their sensorial and emotive experiences can speak to the human suffering they bore witness to, the underlying ageism that permeates our culture, and the social hierarchy that devalues their labor and their worth as they serve on the frontlines during this unprecedented global pandemic.

